# Background Selection From Unlinked Sites Causes Nonindependent Evolution of Deleterious Mutations

**DOI:** 10.1093/gbe/evae050

**Published:** 2024-03-14

**Authors:** Joseph Matheson, Joanna Masel

**Affiliations:** Department of Ecology and Evolutionary Biology, University of Arizona, Tucson, AZ 85721, USA; Department of Ecology, Behavior, and Evolution, University of California San Diego, San Diego, CA 92093, USA; Department of Ecology and Evolutionary Biology, University of Arizona, Tucson, AZ 85721, USA

**Keywords:** linkage disequilibrium, population genetics, nearly neutral theory, forward-time simulation, effective population size, expected heterozygosity

## Abstract

Background selection describes the reduction in neutral diversity caused by selection against deleterious alleles at other loci. It is typically assumed that the purging of deleterious alleles affects linked neutral variants, and indeed simulations typically only treat a genomic window. However, background selection at unlinked loci also depresses neutral diversity. In agreement with previous analytical approximations, in our simulations of a human-like genome with a realistically high genome-wide deleterious mutation rate, the effects of unlinked background selection exceed those of linked background selection. Background selection reduces neutral genetic diversity by a factor that is independent of census population size. Outside of genic regions, the strength of background selection increases with the mean selection coefficient, contradicting the linked theory but in agreement with the unlinked theory. Neutral diversity within genic regions is fairly independent of the strength of selection. Deleterious genetic load among haploid individuals is underdispersed, indicating nonindependent evolution of deleterious mutations. Empirical evidence for underdispersion was previously interpreted as evidence for global epistasis, but we recover it from a non-epistatic model.

**Significance**

As individuals bearing deleterious alleles are removed from a population, other alleles are removed with them, some that are tightly linked near the deleterious allele on a chromosome and some that aren’t linked at all. When the deleterious mutation rate is realistically high, unlinked pairs of loci are a more important influence on the removal of genetic variation. Simulations that assume independent evolution cannot capture removal just by using a lower “effective population size”, because the probabilities of having deleterious alleles on different chromosomes are negatively correlated rather than independent.

## Introduction

The neutral theory of molecular evolution postulates that (i) most genetic diversity observed in natural populations is neutral with respect to an organism's fitness (Kimura 1968; King and Jukes 1969), and (ii) dynamics are well described by models of a single neutral locus in a population of a specified “effective” population size (Ewens 2004; Charlesworth 2009; Masel 2011; Kern and Hahn 2018). A crucial extension of this theory uses a reduction in the effective population size to incorporate the fact that deleterious mutations depress genetic diversity as they are removed, in a process known as background selection (Charlesworth et al. 1993). While the removal of a deleterious allele will depress genetic diversity at neutral loci across the entire genome, neutral loci linked to the deleterious allele will be particularly affected (Charlesworth 2012). Indeed, the effect of unlinked background selection is often considered small enough to be ignored (Charlesworth 2012). Here we revisit this assumption given realistically high genome-wide deleterious mutation rates, by using simulations to evaluate previously derived equations for linked versus unlinked background selection.

In practice, observed genetic diversity at putatively neutral sites is used to estimate the coalescent effective population size $N_{e}$ as the census size of an idealized population that produces the same neutral genetic diversity given demography (Charlesworth 2009) or background selection (Charlesworth et al. 1993). In an idealized population obeying Wright–Fisher or Moran dynamics, genetic diversity depends only on the product of the neutral mutation rate at that locus and the census size of the population (Kimura 1969). Neutral theory considers mutations that either are strictly neutral or so deleterious that they are purged quickly enough as to leave no impact. Nearly neutral theory retains the binary distinction between rapidly purged versus neutral mutations, but allows the ratio of mutations of these two types to vary among species, according to that species’ value of an effective population size (Ohta 1973). Both models assume many independent single loci.

The problem with this binary distinction and independent loci approach is that slightly deleterious mutations are purged only slowly from populations. During this removal process, they depress genetic variation in the genome (Charlesworth et al. 1993). This depression in genetic variation caused by background selection is typically modeled as a decrease in the effective population size for a neutral locus linked to deleterious variants (Hudson and Kaplan 1995; Lohmueller et al. 2011; Comeron 2014). In a population with no recombination and sufficiently large effect deleterious mutations ($N_{e}s\gg 1$), the coalescent effective population size would decrease from $N_{e}$ to $f_{0}N_{e}$, where $f_{0}$ is the equilibrium frequency of individuals with no deleterious mutations, because any neutral variants linked to deleterious variants would be doomed (Charlesworth et al. 1993).

Recombination can decouple neutral variants from deleterious variants, reducing the degree to which background selection depresses neutral variation (Cutter and Payseur 2013). For a single neutral locus linked to a single locus where deleterious mutations with fixed heterozygous effect size sh occur at rate *u* per diploid individual per generation, and with recombination between the loci occurring at rate *r*, heterozygosity at the neutral locus is reduced by a factor $F\approx 1-\frac{ush}{2{\left(sh+r\right)}^{2}}$ (Hudson and Kaplan 1994). This result can be straightforwardly extended to any number of deleterious sites linked to the focal neutral site by assuming (i) that mutation and recombination rates are uniform across a genomic window centered on the neutral site, (ii) that the window is small enough that recombination rate and map length are linearly related, and (iii) that there is linkage equilibrium among deleterious variants e.g. because multiple significantly linked deleterious mutations are not present at the same time (Hudson and Kaplan 1995; Nordborg et al. 1996). In this case, in a genomic window we have


(1) $$\frac{N_{e}}{N}=e^{\frac{-U_{w}}{2sh+R_{w}}}$$

where $U_{w}$ is the total diploid deleterious mutation rate across the entire window, and $R_{w}$ is the map length between the ends of the window (Hudson and Kaplan 1995). Assuming a sufficiently large window size (such that $R_{w}\gg s$), this equation simplifies to


(2) $$\frac{N_{e}}{N}=e^{\frac{-u}{r}}$$

where $\frac{u}{r}$ is the ratio of deleterious mutation rate to recombination rate (Charlesworth 2012). With the same assumptions, similar results can be obtained in the case where both deleterious and beneficial mutations occur (Kim and Stephan 2000).

The results above do not apply across different chromosomes, nor for free recombination between the ends of the window (Hudson and Kaplan 1995). However, deleterious variants do still depress neutral diversity even at unlinked sites: At a bare minimum, a deleterious mutation will eliminate any unique neutral genetic variants in a single individual who dies due to that deleterious mutation. The ratio of $N_{e}$ to *N* with free recombination has been derived as


(3) $$\frac{N_{e}}{N}=e^{-8Ush}$$

where *U* is the total deleterious mutation rate at unlinked loci and sh is the fitness effect of a deleterious allele when in the heterozygous state (Charlesworth 2012).

It has previously been presumed that the reduction in diversity due to unlinked deleterious loci will be much smaller than that from linked loci (Charlesworth 2012). That is, for any given neutral locus, even though *U* across all unlinked deleterious loci is much larger than $U_{w}$ across the window of linked deleterious loci, $\frac{1}{\left(2sh+R_{w}\right)}$ will be so much larger than 8sh that the latter comparison will overwhelm the former comparison, and linked background selection will dominate. Indeed, models of background selection often simply omit the effects of unlinked background selection (Elyashiv et al. 2016; Ewing and Jensen 2016; Torres et al. 2018; McGee et al. 2022).

But omitting the effects of unlinked background selection may be a concern given the sheer quantity of deleterious mutations entering populations, and the greater dependence of Equation (3) on this rate. For example, the average number of new deleterious mutations per human is estimated to be at least 2.1 (Lesecque et al. 2012). This estimate is derived from a point mutation rate of 1.1 × 10^−8^, mutations only being deleterious in the 55% of the 6 × 10^9^ diploid genome that is not dominated by the remnants of transposable elements, which evolves due to this constraint at 94.3% of the rate. This estimate is conservative because some mutations to transposable element regions are deleterious, because more recent estimates of the human point mutation rate are slightly higher at ∼1.341 × 10^−8^ (Wang and Obbard 2023), and because non-point mutations and beneficial mutations are neglected. Some therefore argue that deleterious mutation rates are even higher, closer to ten new deleterious mutations per person in humans (Kondrashov 2017). High deleterious mutation rate estimates are not unique to humans (Haag-Liautard et al. 2007; Popovic et al. 2023).

Here we numerically compare Equations (2) and (3) given human parameters, confirming that unlinked selection has the larger effect. This wouldn’t matter for some purposes, so long as unlinked selection were well described by one-locus models of genetic drift with a lower effective population size. To test this, we perform a multilocus simulation using the fwdpy11 package (Thornton 2014, 2019), which efficiently handles large numbers of non-neutral mutations in relatively large census size populations (Haller and Messer 2017). These simulations agree reasonably well with past analytic results, confirming the importance of unlinked background selection. More importantly, simulations show that deleterious mutations are not well described by one-locus models, with underdispersion of genetic load among haploid genotypes. This pattern of underdispersion has previously been observed empirically for humans and fruit flies, and interpreted as evidence for epistasis (Sohail et al. 2017); we recover it in a non-epistatic model.

## Results

**Fig. 1.**
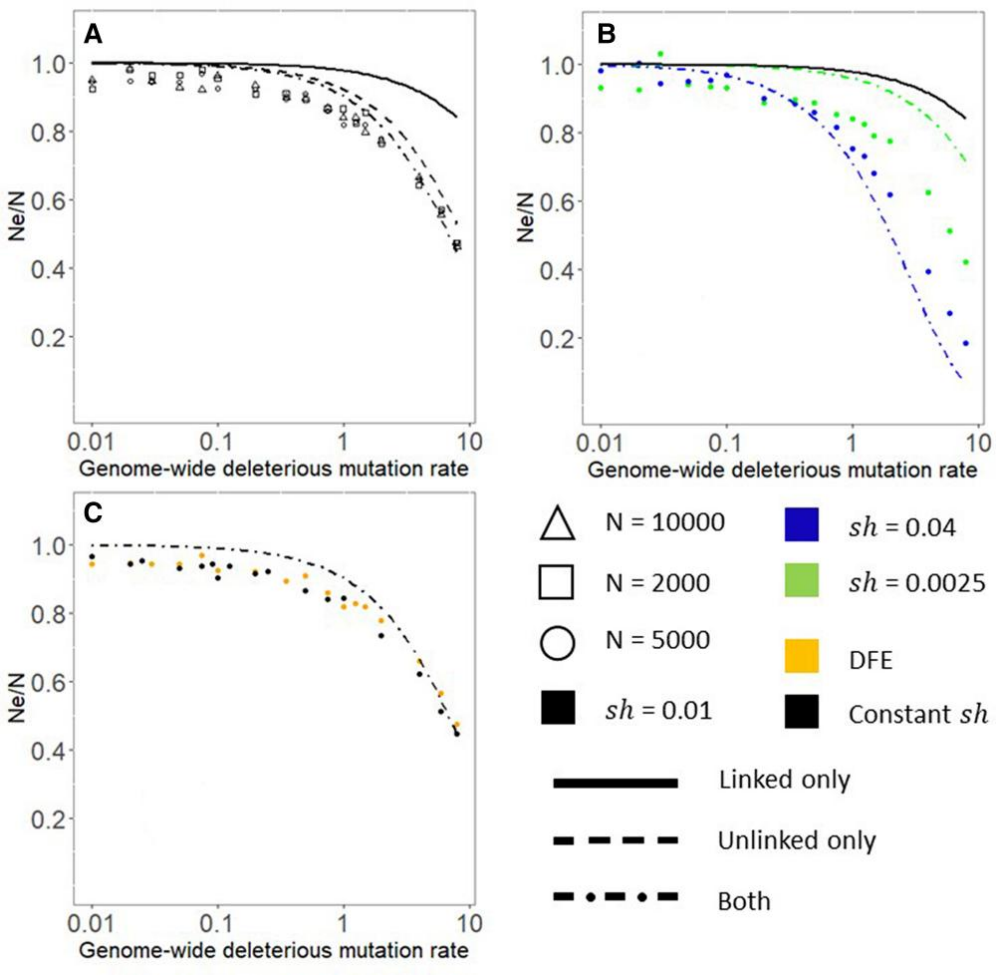
At high deleterious mutation rates, unlinked background selection reduces neutral diversity to a greater extent than linked background selection does. Where not shown, sh=−0.01 and *N* = 5,000. Each point represents a single simulation—we chose to allocate computation to a denser grid of parameter values rather than to replicates of the same parameter values. (a) Unlinked background selection alone (dashed line) is a closer match than linked background selection alone (solid line) to the joint model (dot-dashed line) and to simulations (points). Census population size has no effect on the $\frac{N_{e}}{N}$ ratio across a five-fold change in simulated census population size. (b) Background selection at high mutation rates is stronger with larger selection coefficients, although our joint model exaggerates this effect. The model with linked background selection alone predicts either no or the opposite dependence on sh, and strongly underestimates background selection. (c) The relationship between deleterious mutation rate and $N_{e}$ is similar whether the effect size of new deleterious mutations is constant versus drawn from a distribution of effect sizes with the same mean value of −0.01.

Unlinked background selection reduces neutral diversity more than linked background selection does (Fig. 1a) when the genome-wide deleterious mutation rate *U* is high, specifically when U>1, as is estimated to be the case for humans (Lesecque et al. 2012). This main result is independent of *N* and *s*. A five-fold change in census population size *N* has no significant effect on the $\frac{N_{e}}{N}$ ratio (Fig. 1a). This contradicts models that imply that the impact of background selection is likely to be greater in larger populations (Cutter and Payseur 2013; Corbett-Detig et al. 2015), although deviations from such models have been previously found (Kaplan et al. 1989; Gillespie 2001; Santiago and Caballero 2016).

Larger selection coefficients result in stronger background selection for high *U* but not low *U* (Fig. 1b). This is consistent with a dominant role for unlinked background selection (Eq. 3); linked background selection predicts the opposite effect (Eq. 1). Our approximate analytical joint model (see Materials and Methods) exaggerates the impact of selection strength; for our focal value of sh=−0.01, our joint model slightly underestimates background selection (Fig. 1a and c), distinctly underestimates it for smaller selection coefficients (Fig. 1b), and slightly overestimates it for large selection coefficients (Fig. 1b). The underestimation for smaller selection coefficients has been noted before, and likely occurs because the small coefficients are near a weakly deleterious range, violating the assumption that $N_{e}s\gg 1$ (Johri et al. 2020). A distribution of selective effect sizes behaves similarly to a single sh value with the same mean (Fig. 1c).

**Fig. 2.**
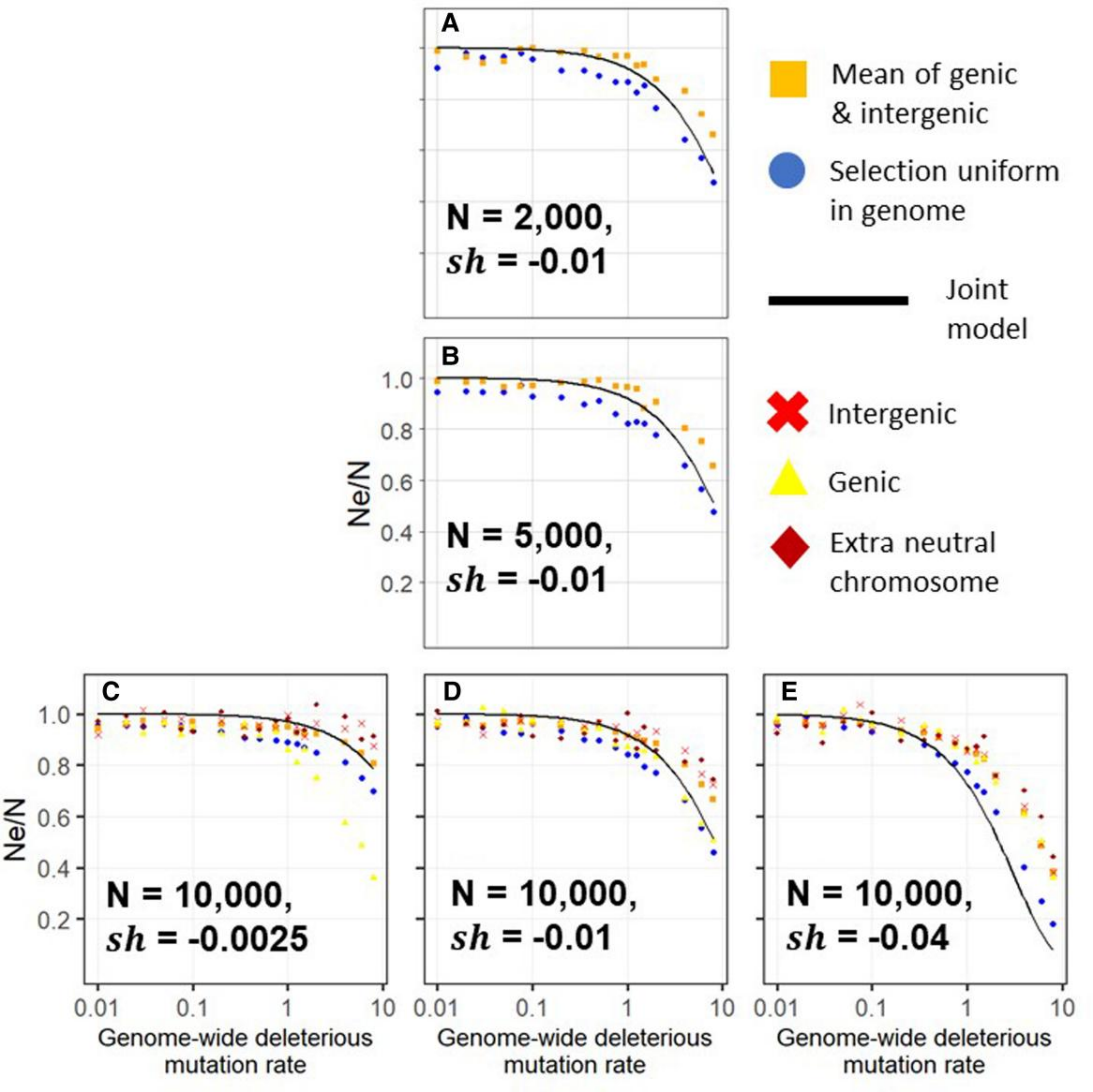
Concentrating deleterious mutations into “genes” slightly weakens overall background selection, and for genic sites, removes the dependence on selection coefficients. As in Fig. 1, background selection becomes significant only at high mutation rates, and census population size does not affect results (a vs. b vs. d). For large selection coefficients, background selection is similar at genic versus intergenic sites (e, yellow triangles vs. red crosses). For small selection coefficients, the gap is considerable (c, yellow triangles vs. red crosses). The dependence on the strength of selection (blue circles and orange squares in c vs. d vs. e, circles match Fig. 1) does not apply to genic sites (yellow triangles in c vs. d vs. e). In all panels, the solid black line is the theoretical expectation incorporating both linked and unlinked background selection, blue circles are simulations where deleterious mutations occur uniformly at random on the genome, and all other shapes are simulations where deleterious mutations are clustered into “genes” (see Materials and Methods). Within the genes condition, sites within genes are shown as yellow triangles, the mean across the genome is shown as orange squares, intergenic sites are shown as red crosses, and sites in an extra chromosome with no deleterious mutations are shown as dark red diamonds.

The simulations above assume that deleterious mutations occur uniformly at random across the genome. A more realistic scenario would be for deleterious mutations to be clustered within a functional subset of the genome. We modify our simulations to model genomes where only 10% of the genome is made up of “genes” subject to deleterious mutations. Concentrating deleterious mutations into more tightly linked “genes” results in a smaller reduction in neutral diversity than found in simulations where deleterious mutations occur across the genome (Fig. 2, orange squares vs. blue circles). This does not depend on population size (vertical comparison on Fig. 2a, b, and d).

Simulations quantify the degree to which neutral diversity is more depressed in genes than in intergenic regions (Fig. 2, yellow triangles vs. red crosses in panels c to e). The reduction in neutral diversity in an extra chromosome which does not experience any deleterious mutations is comparable to the reduction in intergenic regions (Fig. 2, red crosses vs. dark red diamonds in panels c to e), as expected from unlinked background selection. At low mutation rates, there is little depression in neutral diversity, with no appreciable difference between linked versus unlinked sites. There is also little difference with strong selection (Fig. 2e); this is expected as we approach the limit at which each deleterious mutation immediately dooms the genome it appears on (Charlesworth et al. 1993). While larger selection coefficients increase overall background selection (discussed above for Fig. 1 and seen for orange squares and blue circles in Fig. 2c to e), background selection in genic regions is relatively independent of selection coefficient (yellow triangles in Fig. 2c to e) and corresponds well to the joint model for sh=−0.01 (Fig. 2d).

**Fig. 3.**
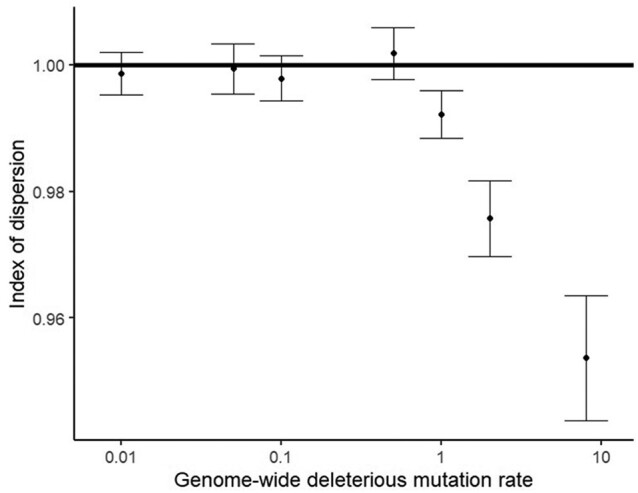
Deleterious mutation rates above 1 create nonindependence among segregating sites. The index of dispersion is calculated as the variance in the number of deleterious alleles per haplotype divided by the mean number of deleterious alleles per haplotype. A haplotype is defined as all the chromosomes from one parent, i.e. each individual contains two haplotypes. A Poisson distribution has an index of dispersion of 1, shown as a horizontal line. Points are the mean index of dispersion from 11 replicate simulations with N=10,000, sh=−0.01, and bars show the standard error in the estimate of the mean.

Strong background selection might be well captured by traditional one-locus models with lower $N_{e}$, if deleterious sites evolve independently. However, at high deleterious mutation rates, linkage disequilibrium occurs (Barton 1998; Barton and Otto 2005), potentially breaking this assumption. To test for independence, we asked whether the variance in the number of deleterious alleles is lower than that expected from the mean under a Poisson distribution (Fig. 3). We find that independent evolution of sites breaks down for U>1. Underdispersion among deleterious alleles has been found empirically for humans and fruit flies (Sohail et al. 2017; Lee 2022) (both with U>1 (Haag-Liautard et al. 2007; Lesecque et al. 2012)). The index of dispersion was 0.94 for missense variants in the human “crucial genome” in which these variants are most likely to be deleterious (Sohail et al. 2017). Note that Sohail et al. (2017) and Lee (2022) interpreted underdispersion as evidence for synergistic epistasis, but we find underdispersion even in a non-epistatic model.

## Discussion

High deleterious mutation rates are well established empirically in humans and a variety of other species. Our simulations confirm previous analytic results, showing that when the deleterious mutation rate is realistically high, unlinked background selection reduces neutral diversity more than linked background selection does. This finding does not depend on the census population size. Background selection is stronger with larger selection coefficients when deleterious sites are distributed uniformly at random, but when deleterious mutations are clustered into genes, this dependence disappears at neutral sites within genic regions. Existing analytical models exaggerate the dependence on selection coefficients, highlighting our incomplete understanding of unlinked background selection. The original view was that high-*s* mutations at sites that are unlinked to the focal site would exclude an effectively random set of individuals in each generation from the effective population size. This heuristic does not easily explain our findings. However, the approximate fit of our simulation results to models suggests that the interference selection regime characterized by Good et al. (2014) is not a good heuristic either.

We focused on the index of dispersion as our genome-wide metric of linkage disequilibrium. There have been recent attempts to disentangle pair-wise measures of linkage disequilibrium from their dependence on allele frequency (Garcia and Lohmueller 2021; Good 2022; Potapova and Kondrashov 2023), and from the complexities of unphased data (Ragsdale and Gravel 2020). It would be interesting for such work to include unlinked controls in future.

Our multilocus simulations omit some population features known to affect neutral diversity (e.g. adaptive evolution (Maynard Smith and Haigh 1974) and temporal changes in population size (Torres et al. 2020)) and ignore variation in others (e.g. in dominance coefficients among deleterious variants (Gilbert et al. 2020) and in recombination rates (Kulathinal et al. 2008)). The purpose of our simulations is to isolate the effects of background selection with high mutation rates, rather than to accurately reflect the genetics of specific populations. Incorporating additional complications into the model might change the quantitative strength of background selection. However, we do not expect adding new complications to change the broader conclusion that unlinked background selection cannot be safely ignored.

It is already known that the effects of linked background selection are critical for explaining differences in neutral genetic diversity among genomic regions, and that failure to do so is a problem for demographic inference (Ewing and Jensen 2016; Pouyet et al. 2018; Johri et al. 2021). Our results bring up the possibility that unlinked background selection could also be important. At low mutation rates (U≪1, see Fig. 3), the effects of unlinked background selection are trivial in magnitude. But at realistically high mutation rates (U>1), unlinked background selection will result in nonindependent evolution among sites. Nonindependence among loci cannot be accounted for with a fudge factor in the effective population size of a one-locus model. In a one-locus model, random changes to allele frequencies are modeled as white noise, i.e. there is no autocorrelation over time. When sites are not independent, being on a bad/good genetic background in one generation will predict being on a bad/good genetic background in the next generation, producing colored noise (Masel 2011). We therefore cannot assume that a one-locus model with lower *N_e_* can accurately capture background selection at unlinked loci.

To save computational cost, previous simulations of background selection treated only a section of a chromosome. The local deleterious mutation rate is set to a value corresponding to high genome-wide *U*, but mutations outside the local window are neglected. We have shown that neglecting them is problematic. This could matter both in the context of demographic inference, and when the goal is to use neutral diversity to distinguish between the effects of linked background selection versus selective sweeps (McVicker et al. 2009; Elyashiv et al. 2016; Murphy et al. 2023). Neglecting unlinked background selection is also a concern for papers investigating only negative selection; e.g. Torres et al. (2018) and Beissinger et al. (2016) look for differences in background selection between human or between maize populations, but their measure of background selection assumes a reference class of neutral sites that are unaffected, to be compared to sites subject to strong background selection.

Another common practice in evolutionary simulations is to rescale parameters (e.g. *N*, *s*, *U*, and *r*) in a manner that keeps products of interest (e.g. *Ns* and *Nr*) constant (Hill and Robertson 1966; Comeron and Kreitman 2002; Hoggart et al. 2007; Kaiser and Charlesworth 2009; Uricchio and Hernandez 2014; Campos and Charlesworth 2019). We found that the strength of background selection is independent of *N*, but is stronger for large *s* (at least outside of genic regions). Reducing *N* while increasing *s* will therefore exaggerate the effect of background selection, i.e. the reduction in $N_{e}$ will be greater than that expected as proportional to the reduction in simulated *N*. The net result of rescaling as it is normally conducted would thus be a reduction in $sN_{e}$ in populations with high *U*.

Accounting for background selection is often conceived as a necessary step prior to inferring demography and/or adaptation. But unlinked background selection in species with high mutation rates globally suppresses neutral diversity to such a degree that it is worthy of scientific attention in its own right. For example, discussions of whether background selection is sufficient to resolve Lewontin's paradox (Buffalo 2021) should consider high *U* and the magnitude of the unlinked background selection that it causes.

To the extent that currently neutral genetic diversity might become relevant in new environments, background selection imposes a genome-wide penalty caused by the constant deluge of deleterious mutations. Massive background selection is thus a sister question to that of overwhelming deleterious mutation load, which has not yet been fully resolved (Kondrashov 1995, 2017; Agrawal and Whitlock 2012; Goyal et al. 2012; Matheson et al. 2023).

## Materials and Methods

### Joint Model

To calculate the expected combined effects of background selection at linked and unlinked sites, we simply multiply together the respective reductions in neutral diversity from Equations (2) and (3). To avoid double counting, we subtract from unlinked selection those mutations that fall within a window of presumed linked selection. Considering constant sh for convenience, this joint model is given by


(4) $$\frac{N_{e}}{N}=e^{-8(U-U_{w})sh}e^{\frac{-u}{r}}$$

where *U* is the total genome-wide deleterious mutation rate. We assume 20,000 windows (this number is chosen to roughly match the number of genes in humans); using different window numbers has minimal impact (supplementary fig. S1a, Supplementary Material online). Results are qualitatively the same if we use a joint model constructed from Equations (1) and (3), with slightly more dependence on window size (supplementary fig. S1b, Supplementary Material online).

### Multilocus Simulations

All simulations were written in Python using fwdpy11 (Thornton 2014, 2019). We simulated populations of *N* diploid individuals undergoing selection against deleterious mutations using a standard Wright–Fisher infinite-sites model for 10*N* generations. Each individual's genome was made up of 23 chromosomes of equal length, with recombination occurring via exactly two crossovers per chromosome, matching data for humans (Pardo-Manuel De Villena and Sapienza 2001).

Deleterious mutations occur with genome-wide rate *U*. In the “no genes” condition, they are located uniformly at random along the chromosomes, while in the “genes” condition they occur only in “genes”. We simulate 1,000 genes, accounting for 10% of the genome, interspersed at regular intervals throughout the genome. Assumptions about “genes” were not chosen to be representative of any particular species, but simply to capture the qualitative consequences of clustering the sites that are subject to deleterious mutations and hence background selection.

For a subset of our simulations, we include a 24th chromosome which experiences no deleterious mutations, to isolate the effects of unlinked background selection. This neutral chromosome is the same length as the original 23 chromosomes and also experiences two crossover events per generation.

A recent study of a large sample of modern European humans estimated a gamma distribution of fitness effects of new non-synonymous mutations with mean $sN_{e}=-224.33$ and $2N_{e}=23,646$, implying a mean sh≈−0.01 (Kim et al. 2017). In our main results, we simplify to use a constant sh=−0.01 to avoid complications from deleterious mutations with $sN_{e}$ so near to 1 that they are effectively neutral. We also explore higher and lower values of *s*, and a gamma distribution with the same mean and shape parameter α=0.169 (Kim et al. 2017). All mutations have h=0.5, and fitness is calculated multiplicatively with no epistasis.

While our forward-time simulations track only deleterious mutations, tree-sequence recording (Kelleher et al. 2018) during the simulation allows neutral mutations to be projected backwards onto the genealogical histories of different genomic regions, enabling us to later compute neutral genetic diversity and hence effective population size. In all simulations, neutral mutations occur uniformly at random on the entire genome at an arbitrary rate $10^{-4}$ per genomic “unit”, for a total rate of 0.23 per genome. This low value provides sufficient resolution of $N_{e}$ at low computational cost. We use msprime (Kelleher et al. 2016) to calculate neutral diversity *θ* on the resulting tree sequence using an infinite-alleles model, and then calculate the effective population size for a simulation using $\theta =\frac{4N_{e}\mu \;}{1+4N_{e}\mu \;}$ and solving for $N_{e}$. When calculating effective population size within genes versus intergenic regions versus extra neutral chromosome (yellow vs. red vs. dark red circles in Fig. 2c to e), we use an infinite-sites model to avoid complications with the distribution of finite neutral sites in genes versus intergenic regions. In these cases, we calculate the effective population size using $\theta =4N_{e}\mu$ and solving for $N_{e}$.

We simulated census population sizes *N* ranging from 2,000 to 10,000. This is compatible with the range of inferred estimates for human effective population sizes (Takahata 1993; Tenesa et al. 2007; McEvoy et al. 2011). We calculate the “observed” value of $\frac{N_{e}}{N}$ from the neutral diversity, to compare with analytical expectations.

## Supplementary Material

evae050_Supplementary_Data

## Data Availability

Simulation code written in Python, and graphs produced with R. Scripts available on GitHub at www.github.com/MaselLab/BackgroundSelection.
