# Supplementary figures and images for: Background Selection From Unlinked Sites Causes Nonindependent Evolution of Deleterious Mutations
Source: Genome Biol Evol. 2024 Mar 14;16(3):evae050. doi: 10.1093/gbe/evae050 (PMC10972689; doi:10.1093/gbe/evae050)

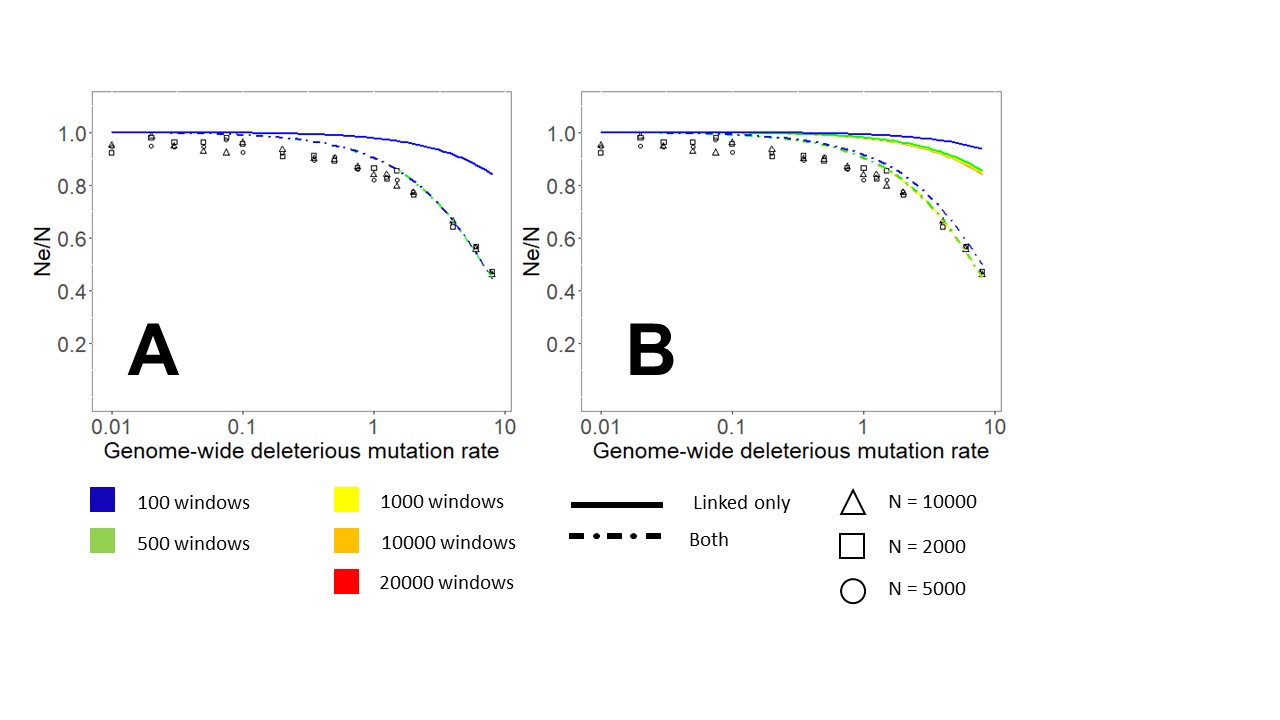

Supplement: evae050_Supplementary_Data [file evae050_supplementary_data.zip › Supplementary Figure 1.jpg]
